# An international comparison of the deinstitutionalisation of mental health care: Development and findings of the Mental Health Services Deinstitutionalisation Measure (MENDit)

**DOI:** 10.1186/s12888-016-0762-4

**Published:** 2016-02-29

**Authors:** Tatiana Taylor Salisbury, Helen Killaspy, Michael King

**Affiliations:** Health Service and Population Research Department, Institute of Psychiatry, Psychology and Neuroscience, King’s College London, De Crespigny Park, London, SE5 8AF UK; Department of Population Health, London School of Hygiene and Tropical Medicine, Keppel Street, London, W1C 7HT UK; Division of Psychiatry, UCL, 6th Floor, Maple House, 149 Tottenham Court Road, London, W1T 7NF UK

**Keywords:** Deinstitutionalisation, Assessment, Mental health services, Validation

## Abstract

**Background:**

Despite its inclusion as a key aspect of successful mental health care service provision by the World Health Organization, there exists a lack of consensus regarding the definition, key components and implementation of deinstitutionalisation. This lack of consensus has also contributed to subjectivity in assessments of countries’ progress towards deinstitutionalisation which act as a barrier to its evaluation and success. In order to provide for reliable within and cross country evaluations of the success of deinstitutionalisation we aimed to develop a quantitative measure of country-level progress towards deinstitutionalisation through the (1) identification of key markers of deinstitutionalisation; (2) development of an assessment tool based on the identified markers; (3) evaluation of the tool’s psychometric properties; and (4) comparison of progress towards deinstitutionalisation across Europe.

**Methods:**

National care standards from 10 European countries and World Health Organization recommendations were used to identify items for the tool. A draft version was reviewed by an international expert panel and assessed for test-retest reliability and internal consistency. Once a final version had been agreed, progress towards deinstitutionalisation was assessed for 30 European countries. We used this opportunity to test convergent validity through comparison with local experts’ assessments. Country total as well as individual item scores were described and compared.

**Results:**

The five-item Mental Health Services Deinstitutionalisation Measure (MENDit) is an objective tool with moderate to very good test-retest reliability (Kappa range: 0.46-1.00) and internal consistency (α = 0.70, 95 % CI 0.25, 0.92). A statistically significant difference between groups was found by one-way ANOVA (*F*(3,26) = 6.77, *p* = 0.002). Post-hoc testing found significant differences between MENDit scores of countries categorised as having advanced levels of deinstitutionalisation and not started or just started. Across Europe, MENDit scores suggest substantial variety in progress towards deinstitutionalisation.

**Conclusions:**

The MENDit has good psychometric properties which support its use in research and as a benchmarking tool to measure national progress towards deinstitutionalisation by policy makers. Across Europe a high proportion of psychiatric beds are still located in psychiatric hospitals. Additionally, low numbers of mental health professionals in many countries may hinder further deinstitutionalisation. These findings corroborate previous mental health systems research and highlight some of the difficulties of deinstitutionalisation.

## Background

During the mid-20^th^ century countries in Western Europe began to shift the locus of mental health care away from mental hospitals to community-based settings. This move to deinstitutionalise mental health care was driven by several factors including growing public awareness of and discomfort towards the poor conditions and human rights violations faced by mental health patients, rising cost of mental hospitals and the introduction of effective psychotropic medication [1, 2]. Despite the move towards deinstitutionalisation throughout much of Europe over the last half century or more, key components of and implementation strategies for deinstitutionalisation are not unanimously agreed [3]. This lack of consensus has made it difficult to evaluate the impact of deinstitutionalisation on clinical, process and structural outcomes. This is especially important as the World Health Organization (WHO) has strongly advocated for deinstitutionalisation over the last decade as a means of improving treatment and care, and upholding the human rights of mental health service users [4–7]. Conversely, critics assert that it does not work and instead leads to increased homelessness and incarceration of people with mental disorders [8, 9].

Research evaluating individual services report significant improvements in clinical and non-clinical outcomes for service users in receipt of community-based care regardless of initial symptom severity [10–13]. However, without a measure of national progress towards deinstitutionalisation, it is difficult to conclude whether deinstitutionalisation works or not. Progress towards deinstitutionalisation has been measured by the number of psychiatric beds [8, 14]. However, numbers of psychiatric beds are only one component of deinstitutionalisation and cannot be used as the sole marker. Recently, the Mental Health Economics European Network asked 31 European mental health experts to describe progress towards the deinstitutionalisation of mental health services in their respective countries as not started, just started, in transition or advanced [15]. However, as no formal criteria were provided for each category, one cannot be certain of their validity.

The aims of this study were to develop a quantitative tool to evaluate progress towards deinstitutionalisation at country-level and conduct an international comparison of progress. To achieve this aim, we sought to address four objectives: (1) to identify key markers of deinstitutionalisation; (2) to develop an assessment tool based on the identified markers; (3) to test the tool’s psychometric properties; and (4) compare progress towards deinstitutionalisation across Europe.

The study is part of ongoing work stemming from the Development of a Measure of Best Practice for People with Long Term Mental Illness in Institutional Care (DEMoBinc) project [16], a collaboration between ten European countries (Bulgaria, Czech Republic, Germany, Greece, Italy, the Netherlands, Poland, Portugal, Spain and the UK). The DEMoBinc project ran from 2007 to 2011 and resulted in the development and validation of the Quality Indicator for Rehabilitative Care (QuIRC). The QuIRC is a measure, completed by facility managers, which assesses the quality of care provided in psychiatric and social care facilities across seven domains: Human Rights; Living Environment; Recovery-based Practice; Social Interface; Self-management and Autonomy; Therapeutic Environment; and Treatments and Interventions.

## Methods

### Identifying the markers of deinstitutionalisation

**Fig. 1:**
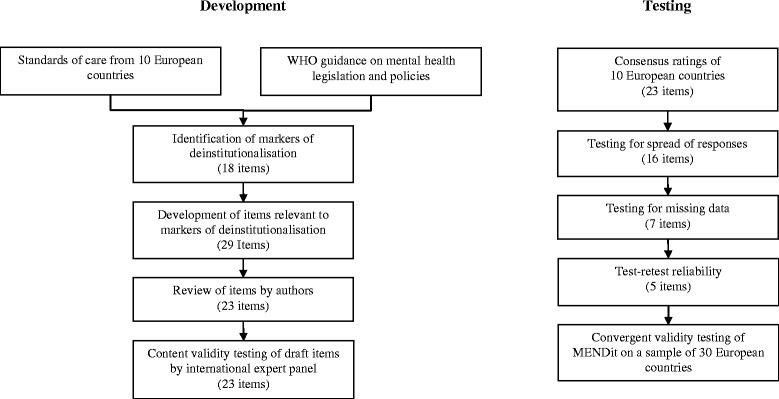
Development and testing of the MENDit

National mental health legislation, policies, plans and programmes from the ten European countries participating in the DEMoBinc project were collated in 2008 (see Fig. 1). In Spain and Germany, national level documents did not exist as services are delivered at the regional level. Therefore, regional documents for Andalusia and Saxony (the states that participated in the DEMoBinc project) were obtained for review. Standards of care, relevant to deinstitutionalisation, common to a minimum of six countries were included as markers of deinstitutionalisation. These identified markers were then supplemented by WHO guidance on mental health legislation and policies [4, 5] to delineate levels of deinstitutionalisation. For example, all countries included the availability of mental health care in primary care services. World Health Organisation recommendations regarding the training of primary care staff in mental health and accessibility of psychotropic medication were included to differentiate between the comprehensiveness of mental health care available in primary care settings.

**Fig. 2:**
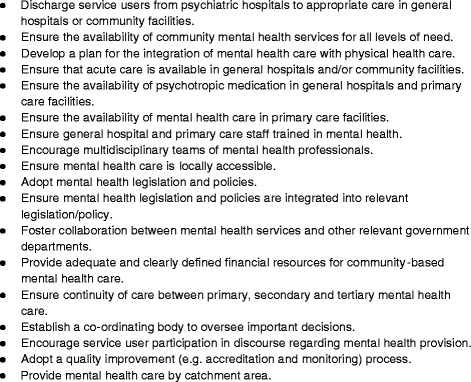
Common markers of deinstitutionalisation

A total of 18 markers of deinstitutionalisation were identified (see Fig. 2). They include the availability and accessibility of mental health care in general hospitals and community facilities, mental health training for medical staff, use of governance procedures, integration of mental health into legislation, policies and plans, and involvement of service users in service planning.

### Development of the deinstitutionalisation tool

A draft tool, based on the identified markers, was developed by TTS. The tool was then reviewed by the other authors to increase compatibility with publicly available data, specifically the WHO *Mental Health Atlas 2005*. The atlas is a compendium of mental health service provision among United Nations member states. It was developed by the WHO through the collation of publicly available data relevant to service provision and corroborated, where possible, by national health departments.

The amended tool was circulated to a 12-member international expert panel to assess content validity. The expert panel comprised: (a) the Principle Investigators for each of the ten countries that participated in the DEMoBinc project, the majority of whom were senior clinical academics with extensive experience in the research and treatment of people with longer term, severe mental health problems and many were renowned nationally/internationally as experts in the field of deinstitutionalisation and the development of community mental health services; (b) a former President of the European Federation of Associations of Families of People with Mental Illness (EUFAMI), a not-for-profit umbrella organisation of European advocacy groups; and (c) a senior member of the WHO Department of Mental Health and Substance Abuse. The text has been amended to include these details. The tool was then used to assess the degree of deinstitutionalisation of mental health care in each of the ten DEMoBinc countries. The authors completed assessments for each country through consensus, rather than independently, informed solely by 2005 Atlas country reports.

### Psychometric testing

The frequency of consensus ratings for each item was assessed to ensure the tool was sensitive to national differences in the provision of mental health care. Items with a restricted range of responses (binary items with more than 90 % of responses in one category, or categorical items with more than two response choices in which more than 80 % of responses fell under a single category) or where at least 30 % of data were missing were excluded. Due to time constraints, consensus ratings were carried out in place of independent ratings. As a result, inter-rater reliability testing was not possible.

Test-retest reliability (carried out by TTS) was assessed using the Kappa coefficient for categorical data. Items which did not reach a moderate level of agreement (Kappa ≤ 0.40) were removed [17]. Remaining items were assessed for internal consistency. A Cronbach’s α of 0.70 or greater was considered indicative of good internal consistency. Following this process, a single score of national deinstitutionalisation was calculated as the sum of all item scores, as further described below.

A one-way ANVOA was used to test for convergent validity. The sum of all item scores (dependent variable) was compared to categorical ratings of progress towards deinstitutionalisation by experts (independent variable) for 30 European countries as reported by the Mental Health Economics European Network [15]. Data from Liechtenstein were excluded as its country profile was not published in the 2005 Atlas. As the number of countries in each Mental Health Economics European Network category varies (range = 2–14), the analysis is only able to provide report trends. All analyses were carried out using STATA, release 12.

## Results and discussion

### Development of the assessment

Twenty-nine items were developed based on the identified markers. A total of seven items were removed from the tool following a review by all authors; five items were omitted because they were incompatible with the data reported in the atlas and two items were combined with other, similar items (items regarding accreditation and monitoring were merged, as were items assessing the introduction and integration of mental health legislation and policies). One item regarding staffing levels (“Staffing levels are adequate”) was deemed to be too subjective and, therefore, amended to obtain the number of mental health professionals (psychiatrists, psychologists, psychiatric nurses and social workers) per 100,000 inhabitants. Availability of community mental health centres/outpatient clinics was split into two separate items (availability of community mental health centres and availability of ambulatory care/outpatient clinics). One item and several response options were amended to improve clarity. The response option, ‘not mentioned’ was added to several items to allow for the recording of missing data. As a result of this process, the tool was reduced to 23 items.The international expert panel’s comments focused on potential subjectivity due to a lack of operational definitions and concerns over the generalisability of the tool across the countries of interest. The potential for subjectivity was addressed by clarifying the text of four items and developing a guide containing operational definitions and descriptions of services. Comments regarding the generalisability of the tool were anticipated as it included questions that were not necessarily reflective of practice across all ten countries. However, given no item was commented upon by more than two expert panel members, no omissions or additions to the tool were made for these reasons.

As it was unclear which items, if any, might be more or less important to deinstitutionalisation, weighting of items was not deemed appropriate. All items were allocated a minimum score of zero and a maximum score of one with a higher score indicating greater deinstitutionalisation. Items with binary response options were coded as 0 and 1; three options as 0, 0.50 and 1; and four options as 0, 0.33, 0.67 and 1.

### Testing of psychometric properties

Seven items were removed from the tool due to their narrow spread of responses (see Table 1). Nine items were excluded due to high levels of missing data which ranged from 30 to 90 %. Two items, availability of community mental health centres (Kappa = 0.32) and availability of vocational and occupational rehabilitation (Kappa = 0.33) were removed from the assessment as a result of poor test-retest reliability. Reliability among the remaining five items ranged from moderate (Kappa = 0.46) to perfect agreement (Kappa = 1.00). The internal consistency of the MENDit was acceptable (α = 0.70, 95 % confidence interval [CI] 0.25, 0.92).

**Table 1:** Items excluded during measure development

Item	Reason for exclusion
Do primary care staff generally receive formal training in mental health (before or after certification)?	Narrow response range
Do general hospital staff generally receive formal training in mental health (before or after certification)?	Missing data
Is psychotropic medication available in general practices?	Narrow response range
Are mental health services provided using clearly defined catchment areas?	Missing data
Availability of day centres	Missing data
Availability of community mental health centres	Poor test-retest reliability
Availability of ambulatory/outpatient care	Missing data
Availability of home care	Missing data
Availability of rehabilitation (vocational, occupational)	Poor test-retest reliability
Availability of crisis teams	Missing data
Availability of specialized mental health services	Narrow response range
Is mental health legislation (laws) in place?	Narrow response range
Is a mental health policy (plan) in place?	Narrow response range
Does mental health policy include a commitment to continuity of care across primary, secondary and tertiary care settings?	Missing data
Do mental health documents include a commitment to the provision of care close to a service user’s place of current/last residence?	Missing data
Have accreditation and service monitoring/auditing systems of mental health facilities been established?	Missing data
Is mental health policy/legislation integrated into other related policy/legislation (e.g. social services, education and employments, justice)?	Narrow response range
Does the government organization responsible for mental health services collaborate (work together) with other relevant government organizations (e.g. social services, education and employments, justice)?	Narrow response range

**Fig. 3:**
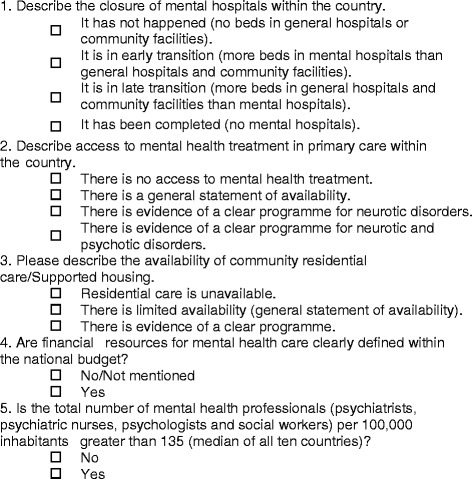
MENDit items

The final version of the MENDit consisted of five items which measure the availability of mental health care outside of mental hospitals (defined as traditional large asylums, not modern, small inpatient mental health units) and resources for the provision of mental health care (see Fig. 3). A country’s total deinstitutionalisation score was calculated as the sum of scores for each item (range = 0–5), with higher scores indicating a greater degree of deinstitutionalisation. Descriptive statistics for 30 European countries are presented in Table 2 and individual item scores in Table 3.

**Table 2:** MENDit descriptive statistics for 30 European countries

Item	Response	Frequency of Response (%)
Closure of mental hospitals	Has not happened	1 (3.33)
	Early transition	20 (66.67)
	Late transition	7 (23.33)
	Completed	2 (6.67)
Mental health treatment in primary care	No access	0 (0)
	General statement of availability	3 (10)
	Clear programme (neurotic disorders)	4 (13.33)
	Clear programme (neurotic and psychotic disorders)	23 (76.67)
Residential care/supported housing	Unavailable	6 (20)
	Limited availability	5 (16.67)
	Evidence of wide availability	19 (63.33)
Financial resources for mental health care	Clearly defined	22 (73.33)
	Not defined/not mentioned	8 (26.67)
Mental health staff per 100,000 inhabitants	>135 (median of all ten countries)	11 (36.67)
	≤135	19 (63.33)
MENDit Total Score: Mean = 3.14, SD = 0.96

**Table 3:** MENDit scores for 30 European countries

Country	Closure of mental hospitals	Primary care treatment	Residential/supported housing	Financial resources	Mental health staff	MENDit score
Austria	0.33	1.00	1.00	0.00	1.00	3.33
Belgium	0.67	1.00	1.00	1.00	0.00	3.67
Bulgaria	0.33	0.33	0.50	1.00	0.00	2.16
Cyprus	0.33	1.00	1.00	1.00	0.00	3.33
Czech Republic	0.33	1.00	0.50	1.00	0.00	2.83
Denmark	0.00	1.00	0.00	1.00	1.00	3.00
Estonia	0.33	1.00	1.00	0.00	0.00	2.33
Finland	0.67	0.67	1.00	1.00	1.00	4.34
France	0.33	1.00	0.00	1.00	1.00	3.33
Germany	0.33	1.00	1.00	1.00	1.00	4.33
Greece	0.67	1.00	1.00	0.00	0.00	2.67
Hungary	0.67	0.67	1.00	1.00	0.00	3.34
Iceland	1.00	1.00	1.00	0.00	1.00	4.00
Ireland	0.33	1.00	1.00	1.00	1.00	4.33
Italy	1.00	1.00	1.00	1.00	0.00	4.00
Latvia	0.33	1.00	1.00	1.00	0.00	3.33
Lithuania	0.33	0.67	0.00	1.00	0.00	2.00
Luxembourg	0.33	1.00	1.00	1.00	0.00	3.33
Malta	0.33	1.00	1.00	1.00	0.00	3.33
Netherlands	0.33	1.00	1.00	1.00	1.00	4.33
Norway	0.33	1.00	0.00	1.00	1.00	3.33
Poland	0.33	0.33	0.50	0.00	0.00	1.16
Portugal	0.67	1.00	1.00	1.00	0.00	3.67
Romania	0.33	0.67	1.00	1.00	0.00	3.00
Slovakia	0.33	1.00	0.50	1.00	0.00	2.83
Slovenia	0.33	1.00	1.00	0.00	0.00	2.33
Spain	0.33	1.00	0.50	0.00	0.00	1.83
Sweden	0.67	1.00	0.00	1.00	1.00	3.67
Turkey	0.33	0.33	0.00	0.00	0.00	0.66
UK	0.67	1.00	1.00	1.00	1.00	4.67

Analysis of the MENDit’s convergent validity resulted in a statistically significant difference between Mental Health Economics European Network categories by one-way ANOVA (*F*(3,26) = 6.77, *p* = 0.002). A positive association was found between MENDit scores and progress towards deinstitutionalisation. Forty-four per cent (ƞ2 = 0.44) of the change in MENDit scores was accounted for by Mental Health Economics European Network category. Post-hoc comparisons using the Tukey-Kramer HSD test found mean MENDit scores were significantly higher in countries with advanced levels of deinstitutionalisation (M = 3.73, SD = 0.74) versus those who had not yet started (M = 1.83, SD = 1.65) and those who had just started (M = 2.56, SD = 0.71, see Table 4). However, the mean score for countries in transition (M = 3.25, SD = 0.42) was not significantly different from the other groups. There was no significant difference between mean MENDit scores of countries that had not yet started and just started deinstitutionalisation.

**Table 4:** Post-hoc comparison of mean MENDit score by Mental Health Economics European Network category

Mental Health Economics European Network category comparison		Difference in mean MENDit score	Std. Err.	Tukey-Kramer t	95 % CI
Advanced	not yet started	1.90	0.57	3.32*	0.33, 3.46
	just started	1.16	0.31	3.71 **	0.30, 2.02
	in transition	0.48	0.43	1.11	-0.70, 1.65
In transition	not yet started	1.42	0.65	2.17	-.38, 3.22
	just started	0.67	0.45	1.53	−0.54, 1.91
Just started	not yet started	0.73	0.59	1.25	-0.87, 2.34

* *p* < 0.05, **p < 0.01

### Deinstitutionalisation of mental health care across Europe

Progress towards the deinstitutionalisation of mental health care varies across Europe with MENDit scores ranging from 0.66 in Turkey to 4.67 in the UK (see Table 3). Twenty-two (70 %) countries reported having a specific budget for mental health. Inpatient psychiatric units operate in all countries except Italy and Iceland. However, it is difficult to know whether these are modern inpatient mental health units or older, large mental hospitals. Sixty-three per cent (*n* = 19) of countries reported providing some degree of community residential care. A clear programme for the provision of mental health care in primary care settings was in place in 77 % (*n* = 23) of countries. A lack of human resources was also identified with nearly two-thirds of countries reporting less than 135 mental health professionals per 100,000 inhabitants (mean = 44 per 100,000).

### Discussion

Despite increasing local and national efforts to deinstitutionalise mental health care, its component parts have not been universally agreed, little research has been undertaken to elucidate the aspects most important to the success of deinstitutionalisation and many countries still do not audit mental health services as rigorously as physical health services. Thus, it is probably no surprise that no quantitative measure of deinstitutionalisation had been developed. We aimed to address this gap in the development of the MENDit. The MENDit provides a transparent and reliable measure of national deinstitutionalisation of the provision of mental health care.

MENDit scores reflect the considerable variation in provision of mental health care across Europe. Many countries have made progress in ensuring access to mental health care through primary care settings and providing community residential care. Nevertheless, greater deinstitutionalisation of care is hindered by a significant reliance on inpatient psychiatric units to provide psychiatric beds and inadequate numbers of mental health professionals to provide comprehensive community-based care. The reduction of psychiatric beds is an important aspect of deinstitutionalisation. In fact, Priebe and colleagues [8] assert that increased numbers of psychiatric beds in settings outside of psychiatric hospitals (e.g. supported housing and forensic facilities) across Europe reflect the ‘reinstitutionalisation’ of people with mental health problems. However, a system with no psychiatric beds is unlikely to be adequate as there will always be individuals who require acute or longer term admission based on the severity of their illness. A truly deinstitutionalised system is one which provides the most appropriate setting (e.g. inpatient, outpatient, hospital, community) and degree of support based on service users’ needs. Although Priebe and colleagues [8] considered supported housing to be a form of reinstitutionalisation, the provision of community based, specialist supported accommodation for people with mental health problems was identified as a common marker of a deinstitutionalised mental health system in this study. Furthermore, the fact that the calculation of psychiatric beds per capita does not strongly correlate with MENDit total scores highlights the complexity of assessing deinstitutionalisation on the basis of bed numbers alone and supports the need for a more comprehensive assessment tool.

### Strengths and Limitations

In keeping with the development of a measure to assess a complex process at the country level, there are several issues which limit its validity. Firstly, there is always the potential for confirmation bias. We sought to reduce this risk by identifying key aspects of deinstitutionalisation through the triangulation of national care standards, expert opinion and WHO recommendations, allocating equal weight to all items in the final measure and using a single source of information to complete country assessments.

The MENDit was designed for compatibility with publicly available information, the WHO *Mental Health Atlas* 2005, due to the challenges in obtaining these data from governments. The WHO *Mental Health Atlas* 2005 was used to complete the assessment despite the availability of updated versions published in 2011 and 2014 [18, 19]. This decision was taken for two reasons. Firstly, the development and testing of the tool began before the updated country profiles were available. Secondly, as convergent validity was tested using Mental Health Economics European Network data published in 2009, it was more appropriate to use the 2005 data for comparison. We appreciate and expect that MENDit scores reported here may not reflect current state of deinstitutionalisation.

The *Mental Health Atlas* represents the most accurate and complete information on national mental health service provision available. However, variation in the consistency and detail of reporting across country profiles has limited the robustness of the measure. Several items which might be regarded as important markers of deinstitutionalisation (e.g. the provision of community-based facilities and mental health training of general hospital staff) were excluded from the MENDit due to substantial missing data.

The lack of detail in the description of settings where psychiatric beds are located led to difficulties in determining progress towards the closure of large, outdated mental hospitals. The *Mental Health Atlas* reports the number of beds located in mental hospitals, general hospitals and other settings which include “private hospitals, military hospitals, hospitals for special populations and long-term rehabilitation centres” [20], p32. As no classification was made in the *Mental Health Atlas*, it was difficult to determine whether the mental hospitals present in all countries were older, traditional mental asylums or modern, inpatient mental health units containing fewer beds with a greater emphasis on treatment and recovery. As a result, all beds in mental hospitals were classed as traditional mental asylums and beds in other settings were classed as modern, inpatient mental health units. However, it is possible that beds in other settings may be more similar to older, traditional asylums or that mental hospitals reflect modern, inpatient mental health units despite their labels.

The growing concern over the rise in numbers of forensic mental health facilities [8, 9] poses a significant barrier to deinstitutionalisation. Although frequently cited by critics of deinstitutionalisation as an example of its failure, forensic psychiatric beds were not mentioned in the standards of care of the countries used to develop the MENDit or WHO guidance. Furthermore, this data is not specifically reported in the *Mental Health Atlas*. It may be inferred that forensic beds fall under hospitals for special populations. However, this distinction is not made in the report. In some countries these beds may fall under the jurisdiction of the judicial system rather than the health system and, as a result, be excluded from the *Mental Health Atlas*. The uncertainty over the reporting of psychiatric beds in forensic settings the features of the settings where psychiatric beds feature may substantially affect MENDit scores. Therefore, the comparison and interpretation of MENDit scores must be cautiously made.

As the MENDit was developed using data from 10 countries, it was not appropriate to conduct more rigorous statistical evaluations of is structure. For example, factor analysis, a common method used to evaluate the factorial validity of an assessment measure which includes items that may be potentially linked to more than one construct [21], is suggested where there are sample sizes of five to ten subjects (in this case countries) per item, with a minimum sample of at least 100 [22]. The brief nature of the instrument also made factor analysis less relevant. Although our sample is small, these 10 countries were purposely chosen to represent varying levels of deinstitutionalisation of mental health care.

Convergent validity tests using a larger sample of 30 countries found agreement between mental health experts’ assessments of countries at early (not yet started and just started) and advanced stages of deinstitutionalisation. However, the MENDit was not able to reflect all four the Mental Health Economics Network categories. It is unclear how much this finding limits the validity of the tool as the categorisation of countries was completed by experts without any operational definitions of each category. Therefore, the validity of their categorisations is questionable. We chose to use this data to evaluate validity due to the lack of any other comparable measure. Furthermore, due to the small numbers of countries included in the analysis, there was insufficient power and this significant finding may not reflect the true level of agreement between MENDit scores and expert ratings. Future research using including a larger number of countries is necessary to conduct more rigorous psychometric testing.

### Implications for research, policy and service provision

Research evaluating individual community services and the effects of the closure of inpatient psychiatric units has indicated that there are significant benefits of community-based care for all service users regardless of symptom severity [10–13]. However, deinstitutionalisation is not simply a bricks and mortar exercise; it also incorporates an environment and ethos conducive to the autonomy and recovery of service users. It is a whole-system process requiring the collaboration and integration of a number of stakeholders, including service users, health and social care providers, policymakers and legislators and must be assessed at the country-level. The MENDit makes large-scale evaluations which examine clinical, social, economic, satisfaction and quality outcomes related to deinstitutionalisation at the country level possible. In addition, the MENDit may also be used at the regional level to identify barriers to deinstitutionalisation which might be addressed through local policy or service improvement planning. For example, a low MENDit score may highlight the issue of deinstitutionalisation for policy makers who, in turn, include deinstitutionalisation priority in mental health policy leading to inclusion of targets and concrete actions through subsequent mental health plans and programmes, respectively. Although items are equally weighted due to a lack of evidence as to the importance of markers of deinstitutionalisation, individual item ratings can be used to identify specific areas where policy and service provision can be improved to meet common benchmarks.

Future research is needed to better understand how changes in mental health provision impact upon levels of deinstitutionalisation across the whole of a country rather than a community or district. This information can then be used to determine the weight allocated to individual MENDit items with the most vital markers of deinstitutionalisation given greater weight. Additionally, improvement in the monitoring, evaluation and reporting of mental health services will likely allow for the inclusion of several excluded items such as continuity of care, presence of an auditing system and training of general hospital staff, which may be useful in differentiating progress towards deinstitutionalisation. Future work should also include an evaluation of the tool’s sensitivity to change using longitudinal data. However, as deinstitutionalisation is usually a slow process, it may take several years before this evaluation can be conducted.

The development of the MENDit has highlighted issues of clarity in the definition of settings where psychiatric beds are located. Future discussions of mental health service provision must address the way in which inpatient mental health units are described to better reflect their environment and ethos. Additionally, the rise of forensic psychiatric settings following the closure of mental hospitals must be acknowledged and their place in modern mental health service provision discussed. The results of these discussions should be incorporated into the operational definitions used by the *Mental Health Atlas* and other reports of mental health systems in order to better assess progress towards deinstitutionalisation.

As the quality of the data improves, further work will be required to amend and re-test future versions of the MENDit. Despite the sole use of European data in the development of the MENDit, the strength of its psychometric properties provides little cause for concern in using it in other countries where deinstitutionalisation is taking place.

## Conclusions

The MENDit provides a quick, yet objective assessment of a country’s level of deinstitutionalisation. This information can then be used to evaluate its relationship with outcomes important to service users, service providers, policymakers and researchers. The five items of the MENDit reflect constructs included in a contemporary definition of deinstitutionalisation: the provision of specific mental health legislation, policies and budgets and the integration of mental health and physical health care systems, which are based on the needs of service users and that focus on the promotion of autonomy. The MENDit has adequate psychometric properties to be recommended for use in the European context, and there is no obvious reason to limit its application in other deinstitutionalising countries. Results of the MENDit indicate varied progress towards deinstitutionalisation among European countries. Significant challenges to deinstitutionalisation include the large number of psychiatric beds located in inpatient psychiatric units and inadequate numbers of trained mental health professionals.

## Abbreviations

CI: Confidence interval
EUFAMI: European Federation of Associations of Families of People with Mental Illness
DEMoBinc: Development of a Measure of Best Practice for People with Long Term Mental Illness in Institutional Care
MENDit: Mental Health Services Deinstitutionalisation Measure
QuIRC: Quality Indicator for Rehabilitative Care
WHO: World Health Organization
